# Discovery of novel dual-target inhibitors of LSD1/EGFR for non-small cell lung cancer therapy

**DOI:** 10.1038/s41401-024-01439-w

**Published:** 2025-01-03

**Authors:** Yu Wei, Ming-ming Sun, Rui-li Zhang, Lin Wang, Li-hong Yang, Chang-liang Shan, Jian-ping Lin

**Affiliations:** 1 State Key Laboratory of Medicinal Chemical Biology, College of Pharmacy and Tianjin Key Laboratory of Molecular Drug Research, Nankai University, Tianjin, 300350 China; 2 Biodesign Center, Tianjin Institute of Industrial Biotechnology, Chinese Academy of Sciences, Tianjin, 300308 China

**Keywords:** LSD1, EGFR, lung cancer, virtual screening, inhibitor

## Abstract

Histone lysine-specific demethylase 1 (LSD1) is overexpressed in various solid and hematological tumors, suggesting its potential as a therapeutic target, but there are currently no LSD1 inhibitors available on the market. In this study we employed a computer-guided approach to identify novel LSD1/EGFR dual inhibitors as a potential therapeutic agent for non-small cell lung cancer. Through a multi-stage virtual screening approach, we found L-1 and L-6, two compounds with unique scaffolds that effectively inhibit LSD1 with IC_50_ values of 6.24 and 9.26 μM, respectively. Using molecular similarity-based screening, 48 analogs of L-1 and L-6 were retrieved from ChemDiv library, 18 analogs were selected for biological activity analysis. Eight compounds showed weaker inhibitory activity against LSD1, with IC_50_ values of 19.79 – 35.70 μM. Moreover, L-1, L-6, and two analogs of L-6 (D-14 and D-16) were found to inhibit triple-mutant EGFR (L858R/T790M/C797S) with potencies ranging from 5.01 to 86.70 μM, and to inhibit double-mutant EGFR (T790M/L858R) with potencies ranging from 2.06 to 64.36 μM. In BaF3 cells that stably express EGFR (L858R/T790M/C797S), the inhibitory activity of L-1, L-6, D-14 and D-16 ranged from 2.72 to 8.99 μM. L-1 that shows the highest biological activity across BaF3 cell, mutant EGFR kinase and LSD1 assays due to its dual targeting of LSD1/EGFR, emerges as a promising lead compound for non-small cell lung cancer treatment. This study demonstrates that L-1 efficiently inhibits lung cancer growth in vitro and in vivo, suggesting it as a potential lead for non-small cell lung cancer treatment, highlighting the utility of virtual screening methods in discovering multi-target inhibitors and strategies for other diseases.

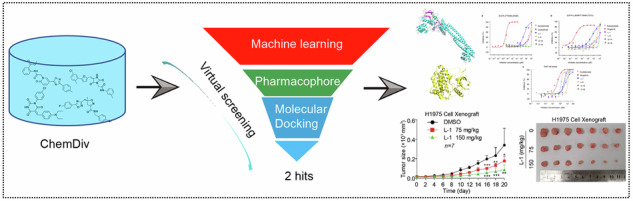

## Introduction

Histone lysine-specific demethylase 1 (LSD1) is a flavin adenine dinucleotide (FAD) dependent protein [1]. It is capable of removing the methylation from both single and double methylated histones H3K4 and H3K9, thereby regulating the interaction between histones and other proteins, and playing an essential role in gene transcription and chromatin structure. Overexpression of LSD1 has been observed in various solid tumors, including non-small cell lung cancer (NSCLC) [2, 3], prostate cancer [4, 5], gastric cancer [6, 7], colorectal cancer [8], bladder cancer [9], esophageal cancer [10], medulloblastoma [11, 12], glioblastoma [13, 14], and breast tumors [15, 16]. Its abnormal expression is closely related to tumor tissue grade, malignancy, and prognosis [17]. In hematological tumors, such as acute myeloid leukemia (AML), LSD1 also plays a crucial regulatory role [18–20].

LSD1 is made up of 852 amino acids arranged into three main segments: the N-terminal SWIRM (Swi3p/Rsc8p/Moira) domain, the C-terminal amine oxidase-like (AOL) domain, and the centrally located Tower domain [21]. The SWIRM and Tower domains provide a scaffold for forming the multiprotein complex, while the AOL domain encompasses both the FAD binding domain and substrate binding domain, which collectively constitute the catalytic active center. The Tower domain consists of two opposing α-helices that are finely tuned and play a critical role in the activity of LSD1. In terms of sequence homology, LSD1 is a close relative of amine-based oxidase, with similarities of 22.4% to polyamine oxidase (PAO) and 17.6% to monoamine oxidase A and B (MAO-A, MAO-B) [22].

In the last decade, significant advancements have been made in the development of effective LSD1 inhibitors [23–27]. Currently, irreversible LSD1 inhibitors regarding tranylcypromine (TCP) as a base are undergoing clinical trials for the treatment of small cell lung cancer (SCLC) and AML. These inhibitors are often used in combination with other drugs like GSK2879552 [28], ORY-1001 [29], INB059872 [23], IMG-7289 [30], JBI-802, phenelzine [31] and ORY-2001 [23]. However, it is important to note that TCP-based LSD1 inhibitors can have side effects resulting from covalent binding to FAD and from high affinity for multiple targets [29]. Furthermore, GSK2879552 has been terminated from clinical trials [32]. Aside from TCP-based inhibitors, irreversible LSD1 inhibitors based on phenelzine [33] and polyarginine [34] also exhibit insufficient activity and poor selectivity. As a result, research into non-covalent LSD1 inhibitors has been on the rise in recent years, with researchers exploring reversible LSD1 inhibitors with different scaffolds [25, 27]. Therefore, reversible LSD1 inhibitors have made significant progress, with pulrodemstat (CC-9001) [35] and seclidemstat (SP-2577) [36] currently undergoing clinical trials. The latest clinical development of LSD1 inhibitors is presented in Table 1 (update to 06/15/2023). Despite the potential for LSD1 inhibitors to be used in cancer therapy, there are currently no LSD1 inhibitors on the market. Thus, there is still an urgent need to develop reversible and effective inhibitors that can produce therapeutic effects.

**Table 1:** Clinical agents as LSD1 inhibitors and their research progress in clinical trials (update to 6/15/2023).

Inhibitor type	Drugs structure	Drugs name	Trial number	Phase	Diseases	Status	Reference
Irreversible		TCP	NCT02273102	Phase I	AML and MDS	Completed	https://classic.clinicaltrials.gov/ct2/show/NCT02273102
			NCT02717884	Phase I/II	Non-M3 AML Blasts	Recruiting	https://classic.clinicaltrials.gov/ct2/show/NCT02717884
			NCT02261779	Phase I/II	Relapsed or Refractory AML	Recruiting	https://classic.clinicaltrials.gov/ct2/show/NCT02261779
		GSK2879552	NCT02034123	Phase I	Relapsed/Refractory Small Cell Lung Carcinoma	Terminated	https://classic.clinicaltrials.gov/ct2/show/NCT02034123
			NCT02177812	Phase I	Acute Myeloid Leukemia	Terminated	https://classic.clinicaltrials.gov/ct2/show/NCT02177812
			NCT02929498	Phase I/II	High Risk Myelodysplastic Syndromes	Terminated	https://classic.clinicaltrials.gov/ct2/show/NCT02929498
		IMG-7289	NCT04254978	Phase II	Essential Thrombocythemia,	Completed	https://classic.clinicaltrials.gov/ct2/show/NCT04254978
			NCT04262141	Phase II	Essential Thrombocythemia, Polycythemia Vera	Recruiting	https://classic.clinicaltrials.gov/ct2/show/NCT04262141
			NCT03136185	Phase II	Myelofibrosis, Post-polycythemia Vera Myelofibrosis (PPV-MF), Post-essential Thrombocythemia Myelofibrosis (PET-MF), Primary Myelofibrosis (PMF)	Completed	https://classic.clinicaltrials.gov/ct2/show/NCT03136185
			NCT04081220	Phase II	Essential Thrombocythemia	Recruiting	https://classic.clinicaltrials.gov/ct2/show/NCT04081220
			NCT02842827	Phase I/II	Acute Myeloid Leukemia, Myelodysplastic Syndrome	Completed	https://classic.clinicaltrials.gov/ct2/show/NCT02842827
			NCT05569538	Phase II	Myelofibrosis	Recruiting	https://classic.clinicaltrials.gov/ct2/show/NCT05569538
			NCT05223920	Phase II	Thrombocythemia, Essential, Primary Myelofibrosis	Active, not recruiting	https://classic.clinicaltrials.gov/ct2/show/NCT05223920
			NCT05558696	Phase II	Polycythemia Vera	Recruiting	https://classic.clinicaltrials.gov/ct2/show/NCT05558696
			NCT05597306	Phase I	Acute Myeloid Leukemia Refractory Acute Myeloid Leukemia Acute Myeloid Leukemia, in Relapse	Recruiting	https://classic.clinicaltrials.gov/ct2/show/NCT05597306
			NCT05191797	Phase I/II	Extensive Stage Lung Small Cell Carcinoma Limited Stage Lung Small Cell Carcinoma	Recruiting	https://classic.clinicaltrials.gov/ct2/show/NCT05191797
		ORY-1001	NCT05546580	Phase I	Acute Myeloid Leukemia, in Relapse, Acute Myeloid Leukemia Refractory	Recruiting	https://classic.clinicaltrials.gov/ct2/show/NCT05546580
			NCT05420636	Phase II	Small-cell Lung Cancer, Neuroendocrine Carcinoma	Recruiting	https://classic.clinicaltrials.gov/ct2/show/NCT05420636
		INCB059872	NCT03132324	Phase I	Sickle Cell Disease	Terminated	https://classic.clinicaltrials.gov/ct2/show/NCT03132324
			NCT03514407	Phase I	Relapsed Ewing Sarcoma	Terminated	https://classic.clinicaltrials.gov/ct2/show/NCT03514407
			NCT02712905	Phase I/II	Solid Tumors and Hematologic Malignancy		https://classic.clinicaltrials.gov/ct2/show/NCT02712905
			NCT02959437	Phase I/II	Solid Tumors, Advanced Malignancies, Metastatic Cancer	Terminated	https://classic.clinicaltrials.gov/ct2/show/NCT02959437
		ORY-2001	NCT03867253	Phase II	Mild to Moderate Alzheimer’s Disease	Completed	https://classic.clinicaltrials.gov/ct2/show/NCT03867253
			NCT04932291	Phase II	Borderline Personality Disorder	Recruiting	https://classic.clinicaltrials.gov/ct2/show/NCT04932291
		JBI-802	NCT05268666	Phase I/II	Locally Advanced Solid Tumor Metastatic Solid Tumor	Recruiting	https://classic.clinicaltrials.gov/ct2/show/NCT05268666
		Phenelzine	NCT03505528	Phase I	Metastatic Breast Cancer	Completed	https://classic.clinicaltrials.gov/ct2/show/NCT03505528
			NCT02217709	Phase II	Adenocarcinoma of the Prostate Recurrent Prostate Cancer Stage I Prostate Cancer	Completed	https://classic.clinicaltrials.gov/ct2/show/NCT02217709
Reversible		CC-90011	NCT04628988	Phase I	Prostatic Neoplasms	Active, not recruiting	https://classic.clinicaltrials.gov/ct2/show/NCT04628988
			NCT04350463	Phase II	Neoplasms	Active, not recruiting	https://classic.clinicaltrials.gov/ct2/show/NCT04350463
			NCT03850067	Phase I	Small Cell Lung Carcinoma	Active, not recruiting	https://classic.clinicaltrials.gov/ct2/show/NCT03850067
			NCT04748848	Phase I	Leukemia, Myeloid	Terminated	https://classic.clinicaltrials.gov/ct2/show/NCT04748848
			NCT02875223	Phase I	Lymphoma, Non-Hodgkin, Neoplasms	Active, not recruiting	https://classic.clinicaltrials.gov/ct2/show/NCT02875223
		SP-2577	NCT03895684	Phase I	Advanced Solid Tumors	Completed	https://classic.clinicaltrials.gov/ct2/show/NCT03895684
			NCT04611139	Phase I	SCCOHT, Ovarian Clear Cell Tumor, Ovarian Endometrioid Adenocarcinoma, Endometrial Cancer	Withdrawn	https://classic.clinicaltrials.gov/ct2/show/NCT04611139
			NCT03600649	Phase I	Ewing Sarcoma, Myxoid Liposarcoma, Sarcoma,Soft Tissue ……	Recruiting	https://classic.clinicaltrials.gov/ct2/show/NCT03600649
			NCT05266196	Phase I/II	Ewing Sarcoma Myxoid Liposarcoma Desmoplastic Small Round Cell Tumor ……	Enrolling by invitation	https://classic.clinicaltrials.gov/ct2/show/NCT05266196
			NCT04734990	Phase I/II	Chronic Myelomonocytic Leukemia-0 Chronic Myelomonocytic Leukemia-1 Chronic Myelomonocytic Leukemia-2 ……	Active, not recruiting	https://classic.clinicaltrials.gov/ct2/show/NCT04734990

NA: The clinical data are not obtained from the ClinicalTrials.gov website, but from the Oryzon website.

Virtual screening (VS) is widely used in the drug discovery process [37]. It helps in identifying lead compounds with new scaffolds and provides structural insights for compound optimization [38–43]. In this study, we utilized a computer-guided approach employing virtual screening to identify novel LSD1/EGFR dual inhibitors as a potential therapeutic agent for lung cancer. First, we employed a multi-stage VS approach, combining random forest (RF), pharmacophore modeling, and molecular docking, to screen a ChemDiv library containing 1,507,829 compounds to discover novel LSD1 inhibitors. A total of 12 compounds were selected and submitted to LSD1 enzymatic assay. Compound L-1 and L-6, characterized by in vitro and in silico experiments, demonstrated their potential as novel LSD1 inhibitors with unique scaffolds. Second, a molecular similarity-based screening of the ChemDiv library identified 48 analogs of L-1 and L-6, among which 18 were selected for biological activity analysis. Out of these, eight compounds demonstrated weaker inhibitory activity against LSD1. Third, multi-target analysis and bioactivity experiments identified that both L-1, L-6 and two analogs of them also possess the potential as EGFR inhibitors, and inhibit triple-mutant EGFR and double-mutant EGFR with varying potency. These four compounds were further tested in BaF3 cell experiments where they demonstrated inhibitory activity. Overall, L-1 exhibited the highest biological activity in LSD1 experiments, mutant EGFR kinase assays, and BaF3 cell experiments. By simultaneously targeting LSD1/EGFR, L-1 emerges as a potential lead compound for the treatment of NSCLC. Finally, we found that L-1 inhibits NSCLC proliferation and tumor growth in EGFR mutant cell-derived xenograft (CDX) mouse model.

## Materials and methods

**Fig. 1:**
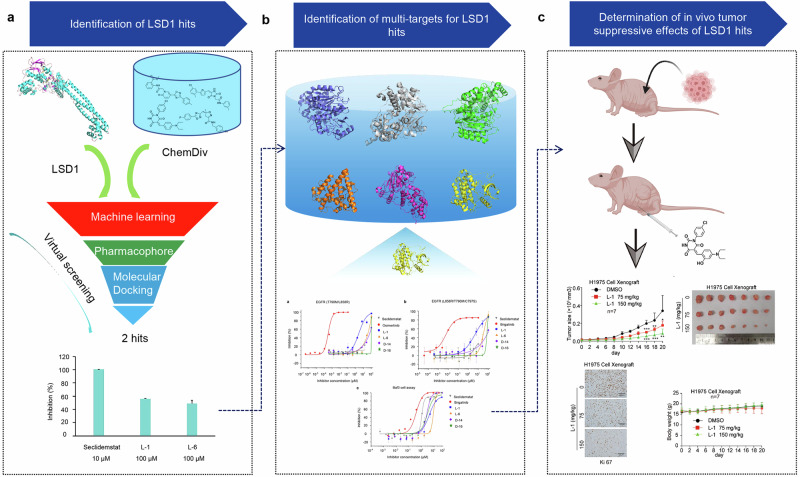
Workflow for identifying a new therapy for lung cancer. **a** Virtual screening of new inhibitors targeting LSD1. **b** Identification of multiple targets for the LSD1 inhibitors. **c** Assessment of in vivo tumor-suppressive effects of the LSD1 inhibitors.

In this study, we present a computer-guided method for the discovery of a new therapy for lung cancer, utilizing a virtual screening approach to identify novel LSD1 inhibitors. The workflow of our study is depicted in Fig. 1.

### Virtual screening protocol

A multistage screening process involving RF model, pharmacophore modeling, and molecular docking method was employed to perform virtual screening of 1,507,829 compounds from the ChemDiv database to identify novel LSD1 inhibitors. For each molecule in ChemDiv library, the ionization and tautomerization states were generated using in Epik at pH 7.0 ± 2.0, with a maximum of 32 stereoisomers using the LigPrep module.

The dataset consisted of 347 inhibitory molecules (IC_50_ < 1 μM) and 289 non-inhibitory molecules (IC_50_ > 10 μM) downloaded from the ChEMBL database [44] and was used to construct the RF models. Molecular descriptors for the molecules in the dataset were calculated using Dragon 6.0 software [45]. Redundant descriptors were eliminated based on criteria including: (1) with too many constant values, (2) a relative standard deviation (RSD) < 0.5%, and (3) a Pearson correlation coefficient >0.95. The dataset were then divided into a training set (80% of the dataset) and a test set (20% of the dataset) using stratified sampling.

RF classifiers [46] were constructed using the scikit-learn library [47], with dragon software-calculated descriptors as input. The RF method, which has been widely used in quantitative structure-activity relationship (QSAR) research, is characterized by robustness in adjustable parameters and high prediction accuracy. The number of trees was set to 1000, and the other parameters were employed using default values. Both internal five-fold cross-validation and an external test set were used to evaluate the performance of the RF models.

The Phase module (Schrödinger, LLC, New York, NY, 2021) implemented in Maestro modeling package was utilized to generate pharmacophore model for LSD1 inhibitors. To create an e-pharmacophore hypothesis, we selected reversible inhibitors CC-90011 and compound 1 (Supplementary Fig. S1) with pyrazolidine-3,5-dione scaffold as the template molecules and were redocked into the substrate-binding pocket and FAD-binding pocket of LSD1 (PDB ID: 6W4K), respectively, using the Glide XP (extra precision) mode with the default settings. The protein structure was extracted from the RCSB Protein Data Bank (PDB), and was preprocessed to remove all crystal water molecules, add hydrogen atoms and missing side chains, and minimize the crystal structure until the root mean square deviation (RMSD) of non-hydrogen atoms reaches ≤0.3 Å with the OPLS_2005 force field using the Protein preparation wizard module in Schrödinger 2021. The pharmacophore hypothesis was generated by utilizing structural and energetic information from Glide XP docking. The generated pharmacophore hypothesis based on CC-90011 and compound 1 are respectively defined as Model I and Model II. Compared to other LSD1 inhibitors like seclidemstat, only CC-90011 has a co-crystal structure with LSD1 (PDB ID: 6W4K). This co-crystal structure provides valuable guidance in virtual screening, particularly in molecular docking, as it helps in assessing the potential affinity between the active molecules and LSD1 more effectively.

The virtual screening workflow in which high-throughput virtual screening (HTVS), standard-precision (SP), and extra-precision (XP) modes were utilized for all molecular docking studies. The energy grid was constructed using the default parameters and the crystal structure (PDB ID: 6W4K). The best 50% of poses generated during the HTVS and SP docking stages were resubmitted to XP docking based on Glide score. The top 25% ranked poses from the XP docking stage were selected for further evaluation. Default settings were employed for all other parameters during grid generation and docking.

### LSD1 enzymatic assay

A total of 12 compounds were purchased from J&K Scientific Ltd. (Shanghai, China). The biological experiments of inhibiting the enzymatic activity of LSD1 by 12 test compounds were conducted by Pharmaron (Beijing, China). Each compound was serially diluted 4-fold in 100% dimethyl sulfoxide (DMSO) from a starting DMSO stock concentration of 20 mM to obtain 10 concentration doses. Seclidemstat was used as a positive control. The diluted compounds were added to the LSD1 assay buffer (50 mM Tris-HCl, pH 7.5, and 1% DMSO) and incubated with LSD1 for 30 min at room temperature. The peptide substrate was then added to initiate the reaction, and the mixture was further incubated for 60 min at room temperature. The reaction was stopped by adding the stop solution, followed by the addition of the detection solution containing protein-A-Eu, streptavidin-d2, and anti-Histone H3 (dimethyl K9) antibody. Fluorescence at Ex/Em = 615/665 nm was measured to determine the half-maximal inhibitory concentration (IC_50_). The IC_50_ was calculated using a nonlinear regression (dose response – variable slope) by fitting the inhibition ratio and log of compound concentrations using Graphpad 8.0.

### SPR binding assay

Surface plasmon resonance (SPR) was employed to evaluate direct binding between compounds and LSD1 using Biacore T200 optical biosensor (Biacore Life Sciences, GE Healthcare). LSD1 was covalently attached to CM5 sensor chip via amine coupling. Binding experiments were conducted at 15 °C in a buffer containing 10 mM HEPES (pH 7.4), 150 mM NaCl, 1% DMSO, and 0.05% Tween, with a flow rate of 30 μL/min. Compounds were serially diluted in the same buffer and injected over the prepared surfaces for 15–25 s at increasing concentrations. Sensorgrams were corrected by subtracting signals from both an untreated reference channel and blank injections. Data analysis was performed using Biacore T200 evaluation software (GE Healthcare).

### Bioactivity test against EGFR

The biological experiments of inhibiting the activity of EGFR by test compounds were also performed by Pharmaron (Beijing). To conduct the kinase assay, the following steps were followed:

a) Transfer compound dilutions into each well of assay plates (784075, Greiner) using Echo 550 system; b) Seal the assay plate and centrifuge compound plates at 1000 × *g* for 1 min; c) Prepare triple-mutant EGFR (L858R/T790M/C797S) and double-mutant EGFR (T790M/L858R) in kinase buffer (5 mM MgCl_2_; 1 mM DTT; 1 mM MnCl_2_ and distilled water), respectively; d) Add 5 μL of triple-mutant EGFR and double-mutant EGFR into 384-well assay plate (784075, Greiner), respectively, and centrifuge the plate at 1000 × *g* for 30 s at room temperature for 10 min; e) Prepare a mixture of TK-substrate-biotin and ATP mixture in kinase buffer; f) Start the reaction by adding 5 μL TK-substrate-biotin and ATP into each well of the assay plate, and centrifuge plates at 1000 × *g* for 30 s; g) Seal the assay plate at room temperature for 40 min; h) Prepare Sa-XL 665 in HTRF detection buffer; i) Add 5 μL Sa-XL 665 and 5 μL TK-antibody-Cryptate into each well of the assay plate; j) Centrifuge plate at 1000 × *g* for 30 s at room temperature for 1 h; k) Read fluorescence signal at 615 nm (Cryptate) and 665 nm (XL665) on Envision 2104 plate reader, and calculate the ratio (665/615 nm) for each well.

% Inhibition is calculated as follow:

$$\% {Inhibition}=\left(1-\frac{{{SINGAL}}_{{cmpd}}-\,{{SINGAL}}_{{ave}_{{-}}{pc}}}{{{SINGAL}}_{{ave}_{-}{vc}}\,-\,{{SINGAL}}_{{ave}_{{-}}{pc}}}\right)\times 100$$

SINGAL_cmpd_ = singal for compound being tested

SINGAL_ave_pc_ = singal for positive controls

SINGAL_ave_vc_ = singal for vehicle controls

The following steps were followed for cell culture and compound treatment:

a) BaF3 cells stably expressing EGFR (L858R/T790M/C797S) were cultured according to ATCC recommended, and only cells in exponential growth phase were used for assays; b) The cells were cultured in 1640 medium, supplemented with 10% FBS and 1% PS; c) The culture was maintained in a humidified incubator at 37 °C with 5% CO_2_; d) The compounds were serially diluted 4-fold from a 10 mM stock solution to obtain 10 doses in DMSO; e) A positive control (10 µM Brigatinib) and 1000 × vehicle control (100% DMSO) were prepared; f) A 150 nL volume of the serial dilution (test compound and positive control) was added to a 384-well cell plate using the Echo system; g) The cells were collected from the flask; h) Only cells with viability greater than 90% were used for the assays; i) 30 µL of the cell suspension (containing 700 cells/well) was dispensed into each well of a 384-well microplate; j) The cells were incubated overnight in a humidified incubator at 37 °C with 5% CO_2_; k) 25 µL of the reagent (CTG) was added to each well and the plates were shaken; l) The plates were incubated at 37 °C in the dark for 30 min; m) The luminescence was read on the Envision plate reader.

% Inhibition is calculated as follow:

$$\% {Inhibition}=\left(1-\frac{{{LUM}}_{{cmpd}}-{{LUM}}_{{ave}_{{-}}{pc}}}{{{LUM}}_{{ave}_{{-}}{vc}}-{{LUM}}_{{ave}_{{-}}{pc}}}\right)\times 100$$

LUM: Luminescence

LUM_cmpd_ = LUM for compound being tested

LUM_ave_pc_ = Average LUM for positive controls (10 μM Control Compound)

LUM_ave_vc_ = Average LUM for vehicle controls (0.1% DMSO)

### Molecular dynamics simulation

In order to investigate the stability of hit compounds within the active site of LSD1 and EGFR, we employed molecular dynamics (MD) simulation on the binding complex of LSD1 and EGFR with hits obtained from molecular docking. The starting 3D structure of LSD1 was obtained from the X-ray crystallographic structure preserved in the Protein Data Bank (PDB ID: 6W4K) at 2.93 Å resolution, while the 3D structure of EGFR was obtained from the X-ray crystal structure (PDB ID: 6LUD) preserved in the Protein Data Bank at a resolution of 2.05 Å. The hits were docked into the crystal structure of the corresponding protein to obtain the protein-compound complex structure. MD simulation was performed using the PMEMD module of AMBER18, with the AMBER FF14SB force field [48] used for proteins and the GAFF force field [49] used for the hits. The binding complex was neutralized by adding sodium or chlorine counterions, and was solvated in a rectangular box of TIP3P water molecules, with a minimal distance of 10 Å between the protein and the box boundary. The system was subject to energy minimization for 10,000 steps. The complex was then gradually heated from 0 to 310 K, followed by equilibration for 5 ns using NVT ensemble, with the protein and ligand constrained using a force constraint of 50 kcal·mol^−1^·Å^−2^. Next, the system was equilibrated for 30 ns using the NPT ensemble, with the constraint force constant gradually decreased and removed for the production MD simulation. The production MD was run at 310 K for 100 ns to obtain a stable MD trajectory. A 12 Å nonbonded interaction cutoff was used during the MD simulation, with the SHAKE algorithm integration used to constrain covalent bonds involving hydrogen atoms, and the particle mesh Ewald (PME) method was applied to treat long-range electrostatic interactions. The frames were saved every 5000 steps for analysis. The binding free energy between the protein and hits was calculated using the MM-PBSA method.

### Cell proliferation assay

Lung cancer H460, H1299, PC9, and H1975 cells were purchased from Procell (Wuhan, China) and cultured in RPMI-1640 medium (Thermo Fisher Scientific, MA, USA) supplemented with 10% fetal bovine serum (FBS, ExCell Bio, Shanghai, China). For cell proliferation assay, the cells were seeded in a 24-well plate (0.5 × 10^4^ cells/well), then treated with L-1 with increasing concentrations (0, 2.5, 5, 10, 20, 40 μM) or vehicle alone. Cell numbers were counted using try-pan blue exclusion on the fourth day.

### Cell-derived xenograft (CDX) models for tumor formation

Nude mice (*nu*/*nu*, female, 4–6-week-old) were purchased from Charles River (Beijing, China) and subcutaneously injected with 2 × 10^6^ H1975 cells harboring on the right flank. Tumor growth was recorded by measurement of two perpendicular diameters using the equation: π/6 × length × width^2^. To explore the effect of L-1 on tumorigenicity in lung cancer, when tumor volumes reached approximately 50 mm^3^, mice received either vehicle control, L-1 (75 mg/kg) and L-1 (150 mg/kg), and they were administered every two days by i.p. injection. The mice were sacrificed, and the tumors were excised, imaged and weighed after inhibitor treatments. Statistical analyses have been done by comparison in relation to the control group with a two-tailed unpaired Student’s *t* test. The animal experiments were performed according to the institutional ethical guidelines approved by the Laboratory Animal Ethics Committee Nankai University.

### Western blot

Cells were lysed with lysis buffer (1.5 M NaCl, 1 M Hepes, 20% NP40, 0.1 M Na pyrophosphate, 0.1 M NaF, 0.1 M Na_3_VO_4_, 0.2 M glycerol phosphate) on ice for 30 min and then centrifuged at 12,000 r/min for 15 min at 4 °C. Protein samples were loaded into 12% SDS-PAGE, then separated by running for different voltage, and transferred onto PVDF membranes (Millipore, MA, USA). The membranes were blocked with 5% non-fat milk for 2 h and then incubated overnight at 4 °C or at room temperature for 2 h with the primary antibody and 1 h at room temperature with secondary antibody. Signals were detected using Luminol substrate solution (Millipore, MA, USA) [50]. The antibodies are listed in Table 2.

**Table 2:** Antibodies used for Western blot analysis.

Antibodies	SOURCE	IDENTIFIER
HK2 Rabbit PolyAb	Proteintech	22029-1-AP
p44/42 MAPK (Erk1/2) Rabbit mAb	CST	9102S
ERK1/2 Mouse mAb	Proteintech	66192-1-Ig
Phospho-Stat3 (Tyr705) (D3A7) P^®^ Rabbit mAb	CST	#9145
STAT3 (124H6) Mouse mAb	CST	#9139
Bata Actin Mouse mAb	Proteintech	66009-1-Ig
Goat anti-rabbit IgG-HRP	Solarbio	SE134
Goat anti-mouse IgG-HRP	Solarbio	SE131
Ki67 Rabbit mAb	CST	12202S
Goat anti-rabbit IgG (H + L) HRP	Bioworld	BS13278

### Immunohistochemical staining

Immunohistochemistry was performed on paraffin-embedded sections. Tissue sections were dewaxed and rehydrated using standard protocol. Antigen retrieval was performed by boiling samples in citrate buffer for 45 min. Endogenous peroxidase activity was inhibited by using 3% hydrogen peroxidase. Sections were blocked in 3% BSA in PBS and incubated in primary antibody (Ki67, CST, MA, USA) overnight at 4 °C. Then the secondary antibody (Goat anti-rabbit IgG (H + L) HRP, Bioworld, OH, USA) was incubated at room temperature for 1.5 h and developed using DAB. Sections were counterstained with hematoxylin.

### Ethics approval and consent to participate

This study was carried out in accordance with the recommendations of Requirements of the Ethical Review System of Biomedical Research Involving Human by National Health and Family Planning Commission of China, Nankai University Laboratory Animal Welfare Ethics Review Committee, Biomedical Ethics Committee of Nankai University with written informed consent from all subjects. All subjects were given a written informed consent in accordance with the Declaration of Helsinki.

### Statistical analysis

Statistical analyses were performed using Student’s *t* test. All data were obtained from three independent experiments performed in triplicate and were presented as the mean ± SD. *P* < 0.05 was considered to indicate a statistically significant difference.

## Results

### Identification of lead LSD1 inhibitors by virtual screening

In this study, we utilized a sequential process of RF modeling, pharmacophore modeling I and II, and a docking scheme to filter the ChemDiv library (containing 1,507,829 compounds) and identify novel and effective LSD1 inhibitors (as shown in Fig. 1). Full details for RF model, pharmacophore modeling, and molecular docking method are provided in the supplementary information. During the construction process of the RF classification model, descriptor filtering was performed based on the importance of descriptors. Different descriptor datasets were used to construct random forest classification models, namely M-10I, M-10II, and M-10III. Compared to M-10I and M-10II, the M-10III model with higher classification performance was initially used to screen the entire ChemDiv library, resulting in 723,083 compounds passing the initial screening. These 723,083 compounds were further screened using pharmacophore models I and II (Supplementary Fig. S2), which resulted in 18,932 compounds and 489 compounds respectively. Third, Glide HTVS, SP, and XP functions were used for molecular docking screening in the virtual screening workflow, leading to a total of 790 compounds and 30 compounds being obtained from the docking screening. The efficacy of each compound was then evaluated by visually inspecting its interaction with the amino acids in the LSD1 ligand binding pocket. Finally, a total of 12 compounds showing promising activity were selected for further in vitro verification of their biological activity against LSD1.

### Biological evaluation targets LSD1

**Fig. 2:**
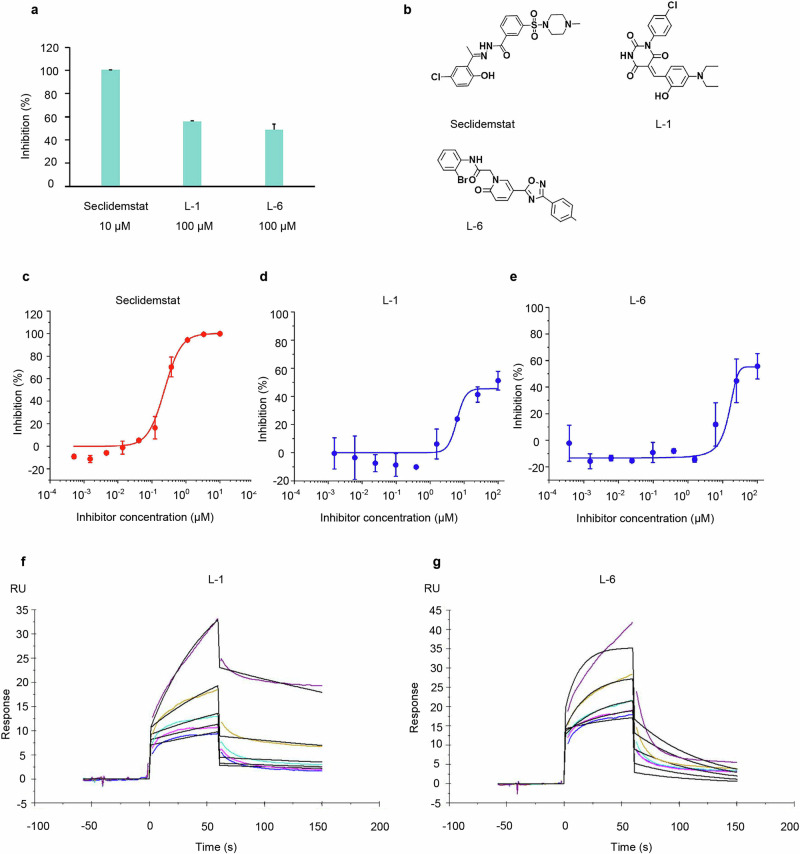
The inhibitory effect of the compound on LSD1. **a** Inhibition of LSD1 enzymatic activity by the potent compounds (100 μM). **b** The chemical structures of seclidemstat, L-1 and L-6. **c** The concentration-response curves of seclidemstat. **d** The concentration-response curves of L-1 and **e** L-6. **f** Surface plasmon resonance sensorgram of the interaction between LSD1 and L-1 and **g** L-6 in 2-fold dilution series.

The biological activity of the selected 12 compounds on LSD1 was evaluated by the LSD1 enzymatic assays. Seclidemstat was used as positive control, which is one of known LSD1 inhibitor in clinical trials, exhibited an IC_50_ value of 0.231 µM in this study (Fig. 2c). Table 3 summarizes the inhibitory potency of 12 compounds (L-1 to L-12) against LSD1. The concentrations for each compound were chosen based on their inhibitory capacities. Seclidemstat, a potent LSD1 inhibitor, showed complete inhibition at 10 μM, while the less potent compounds L-1 and L-6 required 100 μM to achieve significant effects. This approach allowed for a fair comparison of their maximum potential to inhibit LSD1 (Fig. 2a). Among the six compounds identified as FAD-competing inhibitors, L-1 and L-6 exhibited effective inhibitory activity against LSD1 (Fig. 2a), with IC_50_ values of 6.24 μM and 9.26 μM, respectively, while the six substrate-competing inhibitors were found to be inactive. Hence, L-1 and L-6 were seletced for further analysis. The chemical structures of L-1 and L-6 are shown in Fig. 2b, and the concentration-response curves of the L-1 and L-6 in the assay are depicted in Fig. 2d, e, respectively.

**Table 3:** Inhibitory activity of compounds L1 to L12 against human LSD1.

	Compound ID	Bottom	Top	Hillslope	IC_50_ (μM)	Relative potency %	Tc
FAD-competing inhibitors	**L-1**	−4.65	51.52	1.26	6.24	3.7	0.38
	L-2	–	–	–	>100	–	–
	L-3	–	–	–	>100	–	–
	L-4	–	–	–	>100	–	–
	L-5	–	–	–	>100	–	–
	**L-6**	−11.46	55.47	1.86	9.26	2.5	0.30
Substrate-competing inhibitors	L-7	–	–	–	>100	–	–
	L-8	–	–	–	>100	–	–
	L-9	–	–	–	>100	–	–
	L-10	–	–	–	>100	–	–
	L-11	–	–	–	>100	–	–
	L-12	–	–	–	>100	–	–

To further investigate the novelty of the hit compounds (L-1 and L-6), their pairwise similarity with all known LSD1 ligands in ChEMBL and BindingDB was calculated based on the extended connectivity fingerprints (ECFP). The Tanimoto coefficient (Tc) was used to indicate the chemical similarity between two molecules, with values ranging from 0 (completely dissimilar) to 1 (identical). The maximum Tc values of L-1 and L-6 compared to all known LSD1 ligands in ChEMBL are presented in Table 3. As shown in Table 3, the Tc values of L-1 and L-6 are 0.38 and 0.30, respectively, indicating that they are chemically dissimilar to known LSD1 inhibitors. Consequently, the hits obtained from the multi-stage virtual screening possess new chemical structures with low Tc values, and could potentially serve as promising candidates for the inhibition of LSD1 overexpression.

L-1 and L-6 were found to bind within the FAD-binding site of the LSD1 active site, which is distinct from pulrodemstat and seclidemstat, two reversible LSD1 inhibitors currently in clinical trials. These compounds bind to the LSD1 substrate-binding pocket, which is located away from FAD. We conducted Surface Plasmon Resonance (SPR) experiments to verify the reversibility of the binding of compounds L-1 and L-6 to LSD1. The experimental results indicate that L-1 and L-6 displayed specific and reversible binding to LSD1 by SPR (Fig. 2f, g), with dissociation constants (*K*_D_) of 18.25 µM and 26.36 µM, respectively.

### Chemical analogs

**Fig. 3:**
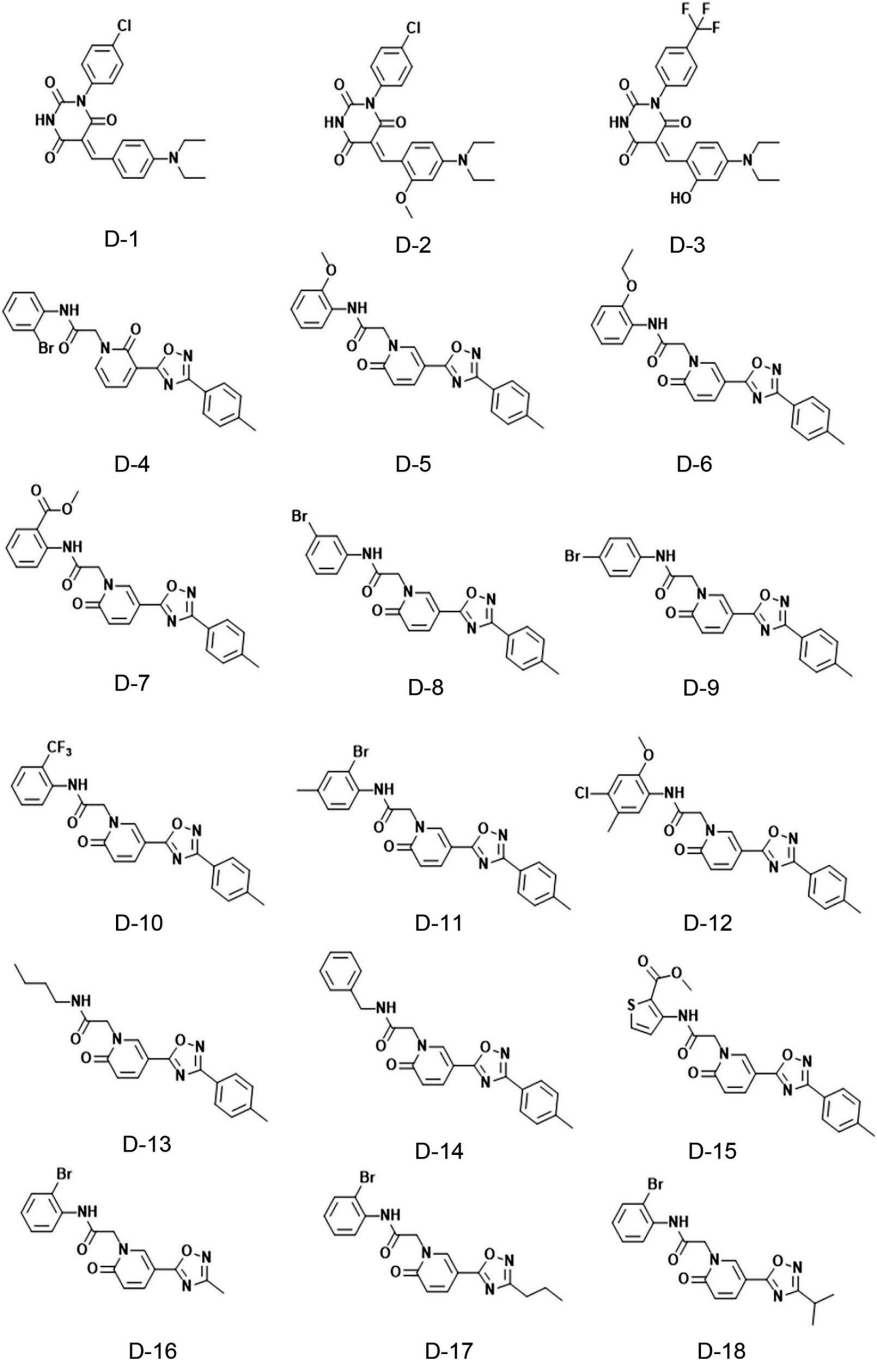
Chemical structures of L-1 and L-6 analogs. Chemical structures of L-1 analogs (compounds D-1 to D-3) and L-6 analogs (compounds D-4 to D-18).

To investigate the inhibitory activity of analogs of L-1 and L-6 against LSD1, a molecular similarity method based on ECFP6 fingerprints was utilized to retrieve analogs of these two hits (L-1 and L-6) from ChemDiv library. A total of 48 molecules were selected, applying a similarity cutoff of 0.6 between the two hits and their analogs. The binding modes of these 48 molecules with LSD1 were further evaluated using molecular docking. Based on the docking score and interaction analysis, 18 molecules were ultimately identified as potential candidates for biological activity analysis. The chemical structures of L-1 analogs (compounds D-1 to D-3) and L-6 analogs (compounds D-4 to D-18) are shown in Fig. 3, and their potency is presented in Table 4. Among the L-1 and L-6 analogs, eight compounds (D-3, D-4, D-5, D-9, D-10, D-14, D-16 and D-17) showed weaker inhibitory activity against LSD1, with IC_50_ values ranging from 19.79 μM to 35.70 μM. Although some of these compounds possess similar scaffolds to L-1 and L-6, they have lower inhibitory activity than L-1 and L-6, the present study provides insights into an effective method for discovering LSD1 inhibitors and reports the inhibitory activity of L-1 and L-6 analogs.

**Table 4:** Inhibitory activity of L-1 analogs (D-1 to D-3) and L-6 analogs (D-4 to D-18) against LSD1.

Compound ID	Bottom	Top	Hillslope	IC_50_ (μM)	Relative potency (%)
**L-1**	−4.65	51.52	1.26	**6.24**	3.7
D-1	–	–	–	>100	–
D-2	–	–	–	>100	–
D-3	8.10	45.78	=3.300	35.70	0.4
**L-6**	−11.46	55.47	1.86	**9.26**	2.5
D-4	5.79	60.26	4.05	29.22	0.5
D-5	7.19	54.06	2.04	21.53	0.7
D-6	–	–	–	>100	–
D-7	–	–	–	>100	–
D-8	–	–	–	>100	–
D-9	3.84	52.57	=3.300	26.61	0.5
D-10	2.43	42.68	=3.300	34.32	0.4
D-11	–	–	–	>100	–
D-12	–	–	–	>100	–
D-13	–	–	–	>100	–
D-14	−1.64	48.09	2.53	28.31	0.5
D-15	–	–	–	>100	–
D-16	−2.33	51.97	=3.300	28.68	0.5
D-17	−0.07	64.23	=3.300	19.79	0.8
D-18	–	–	–	>100	–

The bold values indicate IC_50_ values less than 10 μM.

### Biological evaluation for dual targeting activity

**Fig. 4:**
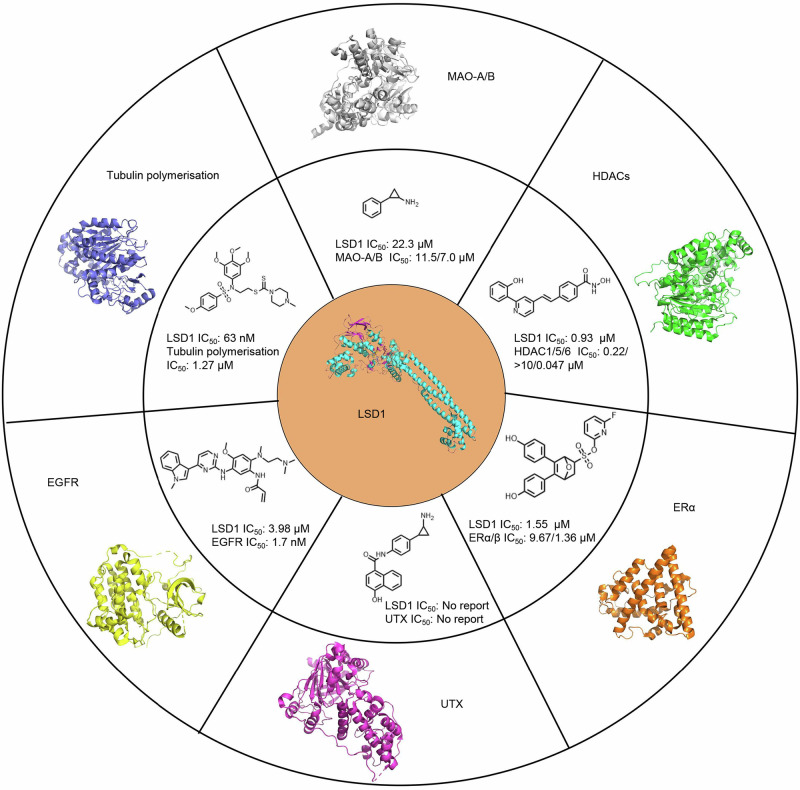
Dual-target inhibitors between LSD1 and multiple protein targets. Crystal structure of LSD1 (PDB ID: 6W4K), MAO-A (PDB ID: 2BXS), HDAC1 (PDB ID: 4BKX), tubulin (PDB ID: 1SA0), ERα (PDB ID: 3ERT), UTX (PDB ID: 6G8F) and EGFR (PDB ID: 6LUD), and dual LSD1/MAO-A/MAO-B, LSD1/HDACs, LSD1/tubulin polymerization, LSD1/estrogen receptor α (ERα), LSD1/X chromosome (UTX), and LSD1/EGFR inhibitors.

Dual-target inhibitors have been proposed as a potential solution to overcome drug resistance and improve potency for the treatment of cancer. Recent studies have demonstrated that dual inhibition of LSD1 and other disease-related proteins can produce synergistic effects and enhance therapeutic efficacy for complex diseases. Dual LSD1/MAO-A/MAO-B [51], LSD1/HDACs [52], LSD1/tubulin polymerization [53], LSD1/estrogen receptor α (ERα) [54], LSD1/X chromosome (UTX) [55], and LSD1/EGFR [56] inhibitors have been reported and exhibited superior clinical effect compared to LSD1 alone [57] (Fig. 4). To explore the potential targets and disease indications for L-1, L-6, D-4, D-5, D-9, D-10, D-14, D-16 and D-17, these compounds were docked into the active site cavity of MAO-A, HDAC1, tubulin, ERα, UTX and EGFR, respectively. The analysis of docking scores and binding poses suggested that L-1, L-6, D-14, D-16 and D-17 possess higher potential binding affinity for EGFR compared to MAO-A, HDAC1, tubulin, ERα and UTX.

**Fig. 5:**
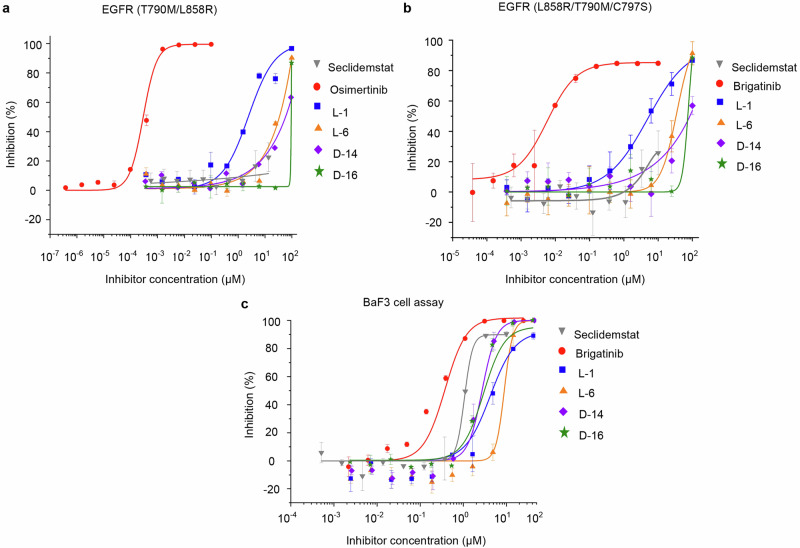
Inhibitory effects of compounds against EGFR mutants and BaF3 cells. **a** The concentration-response curves of four compounds against EGFR (T790M/L858R) and **b** EGFR (L858R/T790M/C797S). **c** The inhibitory effect of four compounds against BaF3_L858R/T790M/C797S.

The potential biological activity of L-1, L-6, D-14, D-16 and D-17 against EGFR was evaluated via kinase assays using triple-mutant EGFR (L858R/T790M/C797S) and double-mutant EGFR (T790M/L858R). Table 5 presents the potency of inhibitors against mutant EGFR. Osimertinib, as a positive compound in the double-mutant EGFR (T790M/L858R), exhibited an IC_50_ value of 0.42 nM, which is consistent with values reported in previous studies (IC_50_ = 1.8 nM) [58]. Brigatinib, as a positive compound in the triple-mutant EGFR (L858R/T790M/C797S) assay, exhibited an IC_50_ value of 5.31 nM, which is consistent with values reported in previous studies (IC_50_ = 2.5 nM) [59]. Among the five compounds tested, four compounds (L-1, L-6, D-14, and D-16) exhibited inhibitory activity against triple-mutant EGFR, with potencies ranging from 5.01 μM to 86.70 μM and also inhibit double-mutant EGFR with potencies ranging from 2.06 μM to 64.36 μM. These four compounds were further tested in BaF3 cell experiments, which utilized cells stably expresssing EGFR (L858R/T790M/C797S), and demonstrated inhibitory activity, ranging from 2.72 μM to 8.99 μM. Brigatinib, as a positive compound in the BaF3 cells harboring EGFR L858R/T790M/C797S mutant assay, exhibited an IC_50_ value of 0.4 µM, which is consistent with values reported in previous studies (IC_50_ = 0.42 µM) [60]. Concentration-response curves are depicted in Fig. 5. Overall, L-1 exhibited the highest biological activity in LSD1 experiments, mutant EGFR kinase assays, and BaF3 cell experiments. We have also conducted experiments on the inhibition of both the double-mutant EGFR (T790M/L858R) and the triple-mutant EGFR (L858R/T790M/C797S) by seclidemstat, as well as experiments on the inhibition at the cellular level in BaF3 cells overexpressing the triple-mutant EGFR (L858R/T790M/C797S). The results have been presented in Fig. 5. We found that seclidemstat does not exhibit inhibitory activity against EGFR double and triple mutants, but it shows good inhibitory activity (IC_50_ = 0.98 μM) in BaF3 cells overexpressing the triple-mutant EGFR (L858R/T790M/C797S). This may be due to the inhibition of LSD1 activity in BaF3 cells by seclidemstat, leading to reduced cell viability. Therefore, unlike the dual-target inhibitors found in this study, seclidemstat has not been confirmed to simultaneously target both LSD1 and EGFR.

**Table 5:** Inhibitory activity of 5 compounds against EGFR mutants and BaF3 cells.

	EGFR kinase assay IC_50_ (μM)		BaF3 cell assay IC_50_ (μM)
Compound ID	T790M/L858R	L858R/T790M/C797S	L858R/T790M/C797S
L-1	2.06	5.01	5.58
L-6	26.52	28.50	8.99
D-14	73.94	86.70	2.45
D-16	64.36	59.57	2.72
D-17	>100,000	>100,000	–

### Molecular modeling exploration

**Fig. 6:**
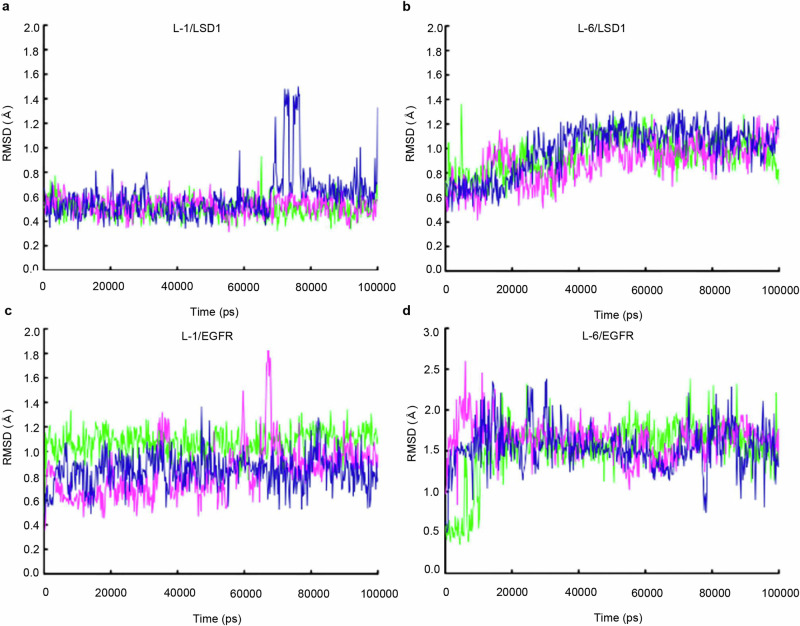
RMSD of molecular dynamics simulations for L-1 and L-6 compounds binding with LSD1 and EGFR. **a** RMSD of L-1 and LSD1, **b** L-6 and LSD1, **c** L-1 and EGFR, **d** L-6 and EGFR.

To investigate the stability of L-1 and L-6 within the active site of LSD1 and EGFR, we performed a 100 ns molecular dynamics (MD) simulation on the binding complex of L-1 and L-6 with LSD1 and EGFR, respectively. Figure 6 displays the calculated root mean square deviation (RMSD) for all four complex systems (L-1/LSD1, L-1/EGFR, L-6/LSD1 and L-6/EGFR), which were within 2 Å, indicating that L-1 and L-6 remains stable in both the FAD binding pocket of LSD1 and the active site of EGFR. Furthermore, the most active compound, L-1, stably bound to the LSD1 and EGFR pockets, with RMSD values of 0.5 Å and 1 Å, respectively. Additionally, we determined the binding free energy of L-1 to LSD1 and EGFR using the MM/PBSA method. The calculated binding affinity values of L-1 to LSD1 and EGFR were −31.31 kcal/mol and −18.90 kcal/mol, respectively.

### L-1 suppresses lung cancer growth

**Fig. 7:**
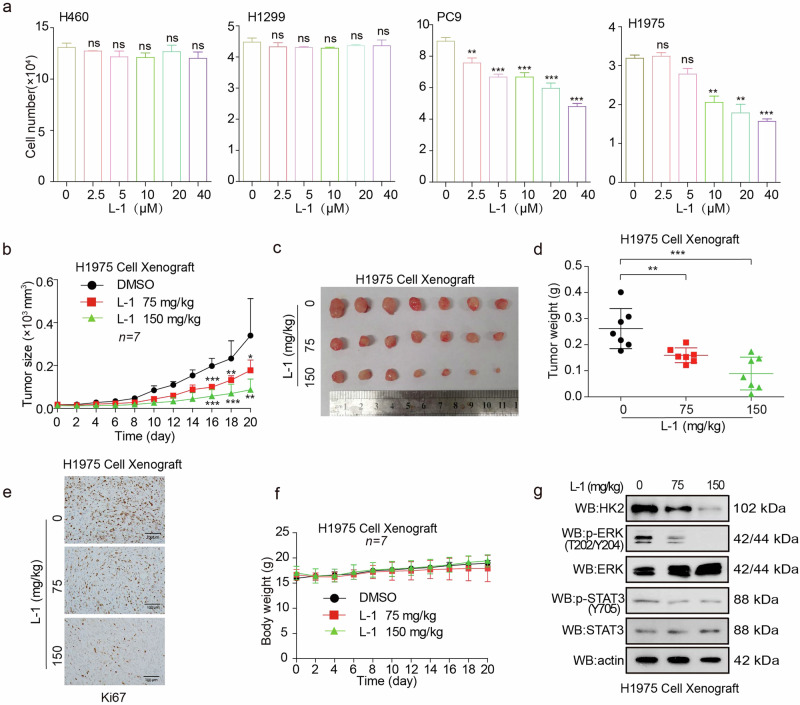
L-1 suppresses lung cancer growth. **a** The cell proliferation was determined by cell number counting assay in lung cancer cells treated with L-1 or DMSO. **b** Tumor growth was compared between xenograft nude mice injected with H1975 cells treated with L-1 or DMSO (*n* = 7). **c** Dissected tumors were shown. **d** Tumor mass of xenograft nude mice injected with H1975 cells and treated with L-1 or DMSO. **e** Ki67 was analyzed in a representative CDX tumor treated with L-1 by IHC. **f** Effects of chronic treatment with L-1 or DMSO on body weights of H1975 xenograft nude mice. **g** Western blot analysis demonstrating the effects of L-1 treatment on various signaling pathway proteins in the H1975 CDX model. The data represent mean values ± SD from three replicates of each sample (*P < 0.05; **P < 0.01; ***P < 0.001).

In order to expolore the anti-cancer activity of L-1 by targeting EGFR mutant, we treated lung cancer cells with L-1 and unexpectedly found that L-1 significantly inhibited PC9 and H1975 (with EGFR mutant) cell proliferation, while not have effect on H460 and H1299 (with EGFR wild type) cell proliferation (Fig. 7a). In the evaluation of the anticancer activity of L-1, osimertinib was selected as the positive control for its well-documented efficacy in inhibiting EGFR-mutant NSCLC cell proliferation. The experimental data for osimertinib, derived from the study by Hu et al. [61], demonstrates its significant inhibitory effect on EGFR-mutant cells, contrasting with its less pronounced impact on EGFR wild-type cells. The cell proliferation experimental results for L-1 are consistent with osimertinib, where we observed a similar pattern of activity, indicating that L-1 may possess comparable inhibitory properties against EGFR-mutant NSCLC cells. Next, we further examined the effect of L-1 in H1975 cells-derived xenograft (CDX) mouse model, and found that H1975 cells tumor with L-1 treatment showed a slower growth rate, smaller tumor size and lighter tumor weight compared to control group (Fig. 7b–d). Furthermore, the expression of Ki67 was examined by IHC. The level of Ki67 was significantly suppressed in the L-1 treatment group (Fig. 7e). However, L-1 treatment did not affect nude the body weight of nude mice (Fig. 7f). We used the dose of 150 mg/kg as the high-dose group for intraperitoneal injection of L-1 in mice, while the dose of the low-dose group was half of the concentration of the high-dose group (75 mg/kg) for the experiment. Indeed, the 75 and 150 mg/kg of L-1 significantly decreased the tumor growth and tumor masses in the H1975 cells-derived xenograft (CDX) mouse model. There was no significant difference in body weight between the drug treatment group and the control group (Fig. 7b–f). Collectively, these data indicated that L-1 suppresses lung cancer cell growth in vitro and in vivo by targeting EGFR mutant. We have detected LSD1/EGFR relevant downstream signaling pathways. The results showed that in animal studies, L-1 could inhibit the LSD1/EGFR relevant downstream signaling pathways (Fig. 7g). In addition, we supplemented the experiment with results showing that L-1 was less effective after LSD1 knockdown in PC9 and H1975 cells compared to control cells (Supplementary Fig. S3).

## Discussion

Multitarget inhibitors have emerged as a promising therapeutic strategy for the treatment of several complex diseases. These inhibitors can simultaneously target multiple pathological processes, resulting in improved therapeutic effects. Some LSD1 inhibitors can act on other targets besides LSD1, including the OBHS-LSD1i conjugate, which exhibited excellent ERα binding affinity and selectivity, and effective inhibitory activity against LSD1 [54]. The multitarget inhibitor MC3324 inhibited both LSD1 and UTX, resulting in significant growth arrest and apoptosis in hormone-responsive breast cancer models, while also causing robust increases in H3K4me2 and H3K27me3 [55]. Osimertinib (AZD9291), a third-generation EGFR tyrosine kinase inhibitor (TKI) approved for the treatment of EGFR-mutated NSCLC, has been characterized as an LSD1 inhibitor with an IC_50_ of 3.98 ± 0.3 μM, and has demonstrated LSD1 inhibition at the cellular level [56].

In this study, we utilized a computational strategy involving virtual screening and bioassays to identify the novel dual LSD1/EGFR inhibitor. Two compounds possessing novel scaffolds were found to inhibit LSD1 with IC_50_ values of 6.24 μM and 9.26 μM, respectively. Using a molecular similarity-based approach, eight analogs were selected for biological activity analysis against LSD1, and were found to exhibit modest activities with IC_50_ values ranging from 19.79 μM to 35.70 μM. Furthermore, our strategy for targeting multiple disease-related proteins simultaneously revealed four compounds with significant inhibitory activity against triple-mutant EGFR and double-mutant EGFR, as validated through BaF3 cell experiments. Among the identified compounds, L-1 demonstrated superior biological activity across all experimental paradigms. The compound’s efficacy was thoroughly evaluated through both in vitro cell proliferation assays and in vivo animal models, providing compelling evidence for its potential therapeutic application. The success of our virtual screening approach not only validates the methodology but also suggests its broader applicability in identifying multi-target inhibitors for various diseases.

This study demonstrates the feasibility of rational design in developing dual-targeting compounds, potentially opening new avenues for treating complex diseases. The computational approach we employed could be adapted for other target combinations, potentially accelerating the discovery of novel therapeutic agents. Moreover, the identification of compounds with dual LSD1/EGFR inhibitory activity provides new insights into the development of more effective cancer treatments, particularly for cases where single-target therapies have shown limited efficacy or led to resistance.

The virtual screening approach used in this study could revolutionize drug discovery and development processes by:

i. Accelerating the identification of novel therapeutic candidates;
ii. Enabling more personalized treatment approaches;
iii. Facilitating the development of resistance-resistant therapies;
iv. Supporting the rational design of combination therapies;

Moreover, the integration of artificial intelligence and structure-based drug design (SBDD) with our virtual screening approach could further enhance the efficiency and accuracy of multi-target drug discovery, potentially leading to more effective therapeutic options for patients with complex diseases.

## Supplementary information

Supplementary information
