# Supplementary material for: Discovery of novel dual-target inhibitors of LSD1/EGFR for non-small cell lung cancer therapy
Source: Acta Pharmacol Sin. 2025 Jan 3;46(4):1030–44. doi: 10.1038/s41401-024-01439-w (PMC11950244; doi:10.1038/s41401-024-01439-w)
Supplement: Supplementary file 1 — Supplementary information [file 41401_2024_1439_MOESM1_ESM.docx]

# Supporting information

**Figure legends**

**Figure S1. a** 2D chemical structure of CC-90011 and **(b)** compound 1.

**Figure S2. a** The docking binding modes of CC-90011 and compound 1 **(b)**. Compounds are shown in pink sticks, the residues are shown in cyan sticks. H-bonds are shown in yellow dashed line. **c** The pharmacophore model I and model II **(d)**.

**Figure S3.** The cell proliferation assays for non-small cell lung cancer (NSCLC) cell lines PC9 and H1975, assessing the efficacy of the compound L-1.


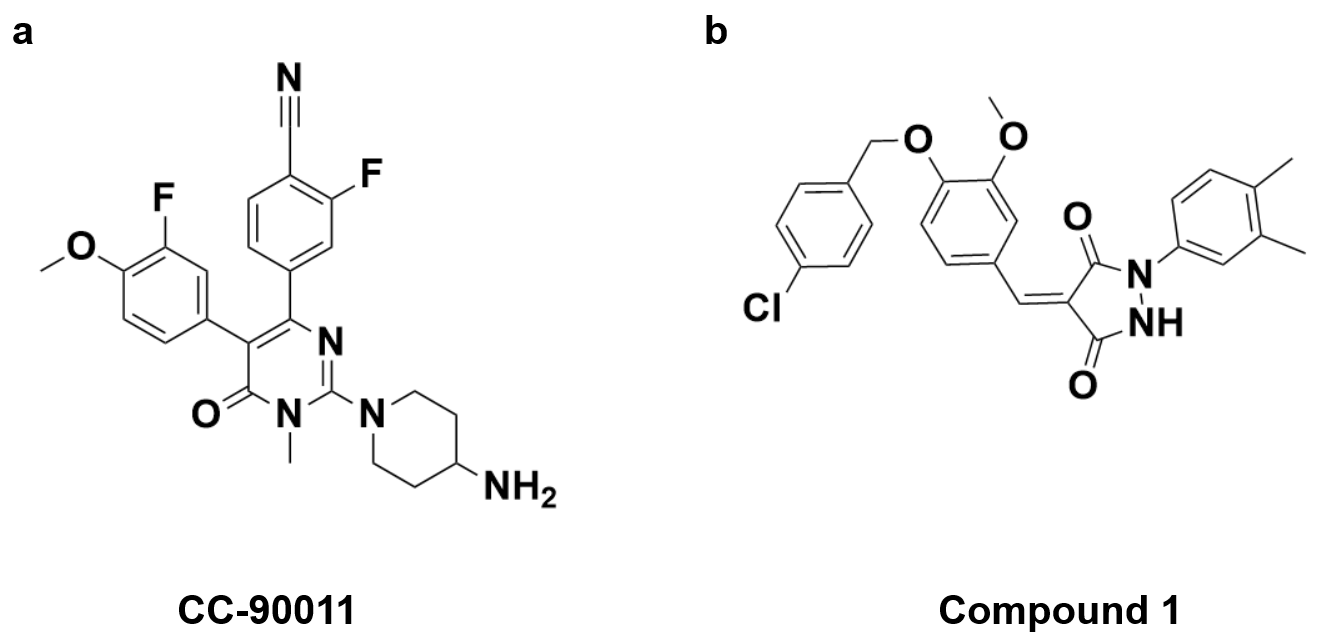


**Fig. S1 a** 2D chemical structure of CC-90011 and **(b)** compound 1.

#### 1. Establishment and validation of RF model

The molecular data was randomly split into training (80%) and test (20%) sets. RF models were built using training set, and the performance of the models were evaluated by the test set. Initially, the data set D-10I has 450 descriptors which were used to construct the M-10I model. Then, the importance of descriptors used in the M-10I model was estimated by calculating the average decrease of the Gini coefficient of each descriptor (MeanDecreaseGini), so as to study the influence of the number of descriptors on the classification ability of the model. MeanDecreaseGini index is used to remove unimportant descriptors from the M-10I model. The unimportant (MeanDecreaseGini <2) descriptors were removed from the 450 descriptors, and the remaining 23 descriptors were used to construct the M-10II model. Then, the importance of 23 descriptors of model M-10II is evaluated, and 10 descriptors with MeanDecreaseGini greater than 10 are reserved for building M-10III.

The molecular data (training set: 80%, test set: 20%) were imported into the framework of random forest model and run. After descriptors preprocessing, the performance of three random forest models were evaluated by sensitivity (SE), specificity (SP), total accuracy (Q), Matthews correlation coefficient (MCC) and area under receiver operating characteristic curve (AUC). Then, the classification ability of the models M-10I, M-10II and M-10III was evaluated, and the evaluation indexes were shown in the **Table S1**.

**Table S1. Evaluation indexes of models M-10Ⅰ, M-10Ⅱ and M-10Ⅲ.**

| **Model** | **Number of descriptors** | **TP** | **FP** | **TN** | **FN** | **SE** | **SP** | **Q** | **MCC** | **AUC** |
| --- | --- | --- | --- | --- | --- | --- | --- | --- | --- | --- |
| M-10I | 450 | 56 | 6 | 48 | 4 | 0.933 | 0.889 | 0.912 | 0.824 | 0.973 |
| M-10II | 23 | 55 | 6 | 48 | 5 | 0.917 | 0.889 | 0.904 | 0.806 | 0.977 |
| M-10III | 10 | 56 | 6 | 48 | 4 | 0.933 | 0.889 | 0.912 | 0.824 | 0.974 |

According to the **Table S1**, the index SP (0.889) of model M-10III is the same as that of models M-10I and M-10II, but the index SE (0.933) of model M-10III is the same as that of model M-10I and higher than that of model M-10II, indicating that models M-10III and M-10I have stronger discrimination ability to positive molecules. The Q (0.932) and MCC (0.824) of model M-10I are the same as those of model M-10III, but the AUC (0.973) of model M-10I is lower than that of model M-10III. In general, model M-10III has better classification performance than M-10I and M-10II, and considering the computational cost and time, model M-10III established with 10 descriptors is selected for the first step of virtual screening.

#### 2 Generation of pharmacophore model

To mimic the key interactions between compounds and proteins, reversible inhibitors CC-90011 and compound 1 with pyrazolidine-3,5-dione scaffold were redocked into the substrate-binding pocket and FAD-binding pocket of LSD1 (PDB ID: 6W4K), respectively. For CC-90011, it binds on the LSD1 substrate-binding pocket via a salt-bridge with Asp555, hydrophobic contacts with a hydrophobic pocket formed by Trp695, Ile356, Leu677, Leu692, and Phe358, and an H-bond with catalytic Lys661 (Supplementary **Fig. S2A**). For compound 1, two carbonyl groups of the pyrazolidinedione moiety form a hydrogen bond network with residues Ser289 and Arg316, and its amino group forms another strong hydrogen bond with Thr624 (Supplementary **Fig. S2B**). The docking pose that determined and scored by Glide XP, was consistent with reports by Sun et al.. Based on the interactions reported in the literature and molecular docking results, we constructed the pharmacophore model I and II (Supplementary **Fig. S2C and S2D**), respectively. As shown in Supplementary Fig. S2B and S2D, the generated pharmacophore hypothesis based on CC-90011 includes one hydrogen bond acceptors (A), one positively charged group (P), one hydrophobic group (H) and two aromatic ring (R) (Model I), and the generated pharmacophore hypothesis based on compound 1 includes two hydrogen bond acceptors (A), one hydrogen bond donor (D), and two aromatic rings (R) (Model II).


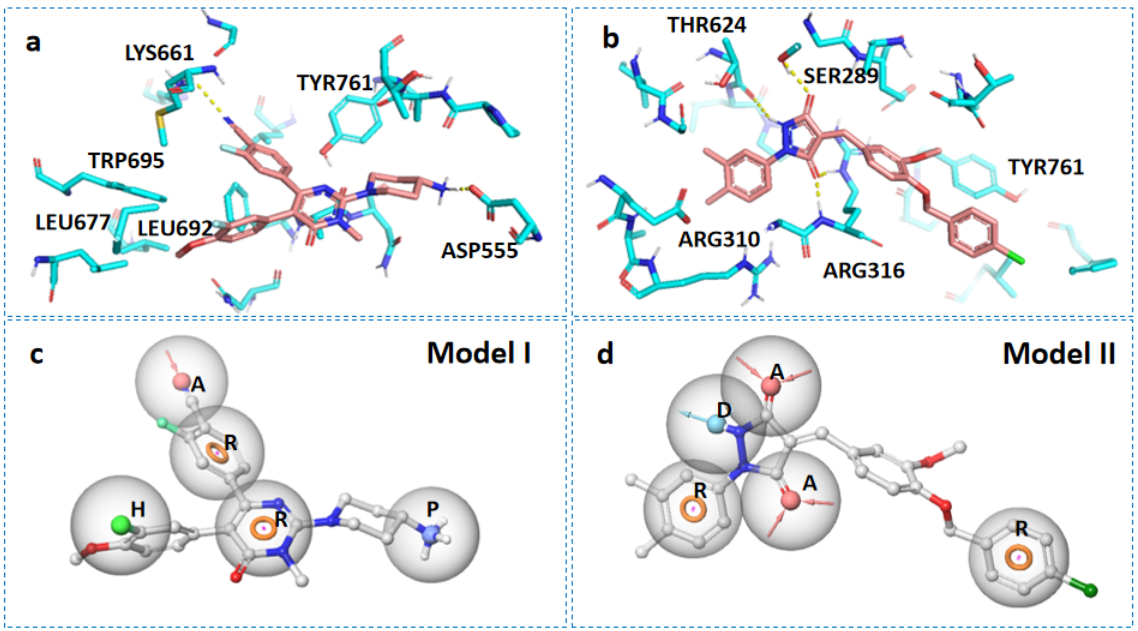


**Fig. S2 a** The docking binding modes of CC-90011 and compound 1 **(b)**. Compounds are shown in pink sticks, the residues are shown in cyan sticks. H-bonds are shown in yellow dashed line. **c** The pharmacophore model I and model II **(d)**.

#### Molecular docking

In order to evaluate the accuracy of molecular docking, CC-90011 and FAD were extracted from the LSD1 co-crystallized complex (PDB ID: 6W4K). CC-90011 and FAD were respectively redocked to the substrate-binding pocket and FAD-binding pocket of LSD1 using three docking schemes, which includes high-throughput virtual screening (HTVS), standard precision (SP) and extra-high precision (XP) with the default docking parameters. Supplementary **Table S2** lists the root-mean-square deviation (RMSD) values between the redocked and crystallized conformations of FAD and CC-90011. Noteworthy, CC-90011 and FAD with RMSD values below 2 Å are accepted, which indicates that HTVS, SP and XP are reliable methods for docking research in this study.

**Table S2 Docking scoring and RMSD for three different docking protocols.**

|  | **CC-90011** | | **FAD** | |
| --- | --- | --- | --- | --- |
| **Docking protocol** | **Docking score (kcal/mol)** | **RMSD (Å)** | **Docking score (kcal/mol)** | **RMSD (Å)** |
| HTVS | -7.685 | 1.429 | -16.995 | 1.1239 |
| SP | -7.992 | 0.492 | -18.796 | 1.1592 |
| XP | -7.574 | 1.102 | -21.099 | 0.6908 |


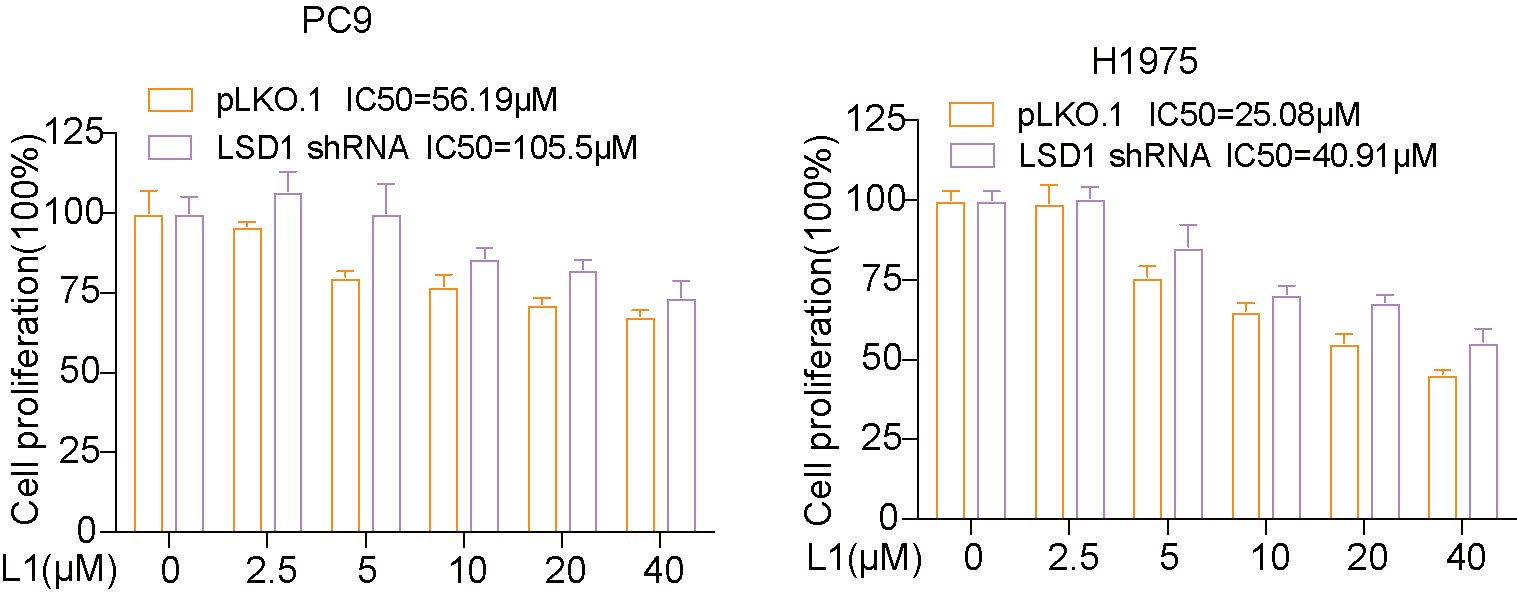


**Fig. S3** The cell proliferation assays for non-small cell lung cancer (NSCLC) cell lines PC9 and H1975, assessing the efficacy of the compound L-1.
